# An Internet-based discussion forum as a useful resource for the discussion of clinical cases and an educational tool

**DOI:** 10.4103/0970-0358.73448

**Published:** 2010

**Authors:** Deborah P. S. Foong, Duncan A. McGrouther

**Affiliations:** Department of Plastic Surgery and Burns, New Queen Elizabeth Hospital Birmingham, Mindelsohn Way, Birmingham, B15 2WB, UK; 1 University Hospitals South Manchester, Southmoor Road, Manchester, M23 2 LT, UK

**Keywords:** Discussion, education, forum, internet, yahoogroup

## Abstract

**Introduction:**

An Internet-based group of plastic surgeons was formed in India in February 2001. It has 1290 members and seeks to facilitate online discussion. These discussions were reviewed to assess their value in education and aiding patient management.

**Materials and Methods:**

All messages and discussions between August 2007 and July 2008 were examined retrospectively. Data were collected regarding topics, replies, and use of clinical images.

**Results:**

A total of 2217 messages were exchanged within 330 separate discussions (mean = 6.7 messages per discussion, range = 0–45). A total of 164 discussions contained photographs (50%). Mean number of photographs per discussion was five (range = 0–34). Discussions included requests for advice on complex cases (40%), interesting cases and their management/outcome (25%) and courses/conferences (30%). Topics discussed include training/courses (26.7%), cleft (15.4%), aesthetics (13.1%), trauma (12.5%), head and neck (8.4%), cutaneous (6.4%), perineal/genital reconstruction (6.1%), and scar management (4.7%).

**Discussion:**

Forums like this facilitate discussion between individuals in remote locations. They provide easy access to the expertise of a large cohort of highly experienced surgeons. Most discussions were clinical, involving challenging situations. The discussions are open and nonjudgmental, hence encouraging contribution and healthy debate. We encourage its use as an educational tool and a platform for discussion.

## INTRODUCTION

In February 2001, an Internet-based group was formed by plastic surgeons in India. Its purpose was to facilitate discussion and exchange of ideas and information between plastic surgeons. It is aptly called “plastic_surgery@yahoogroups.com”. Membership is by invitation and limited to consultants and trainee plastic surgeons. To assess its usefulness as an educational tool, platform for discussion and channel for exchange of ideas, all discussions from September 2007 to September 2008 were reviewed.

## MATERIALS AND METHODS

All messages and discussions posted within the Internet-based discussion group from September 2007 to September 2008 were examined. Data regarding the types of discussions, topics, replies, and use of images were collected.

## RESULTS

During the 12 months from September 2007 to September 2008, 2217 messages were posted. This occurred within 330 discussion threads, giving an average of 6.7 messages per thread (range = 1–45). The types of discussions included ones asking for advice/ideas on treatment (40%), disseminating information on meetings/courses/fellowships (30%), case reports (25%) and introducing new members (4%).

**Figure 1:**
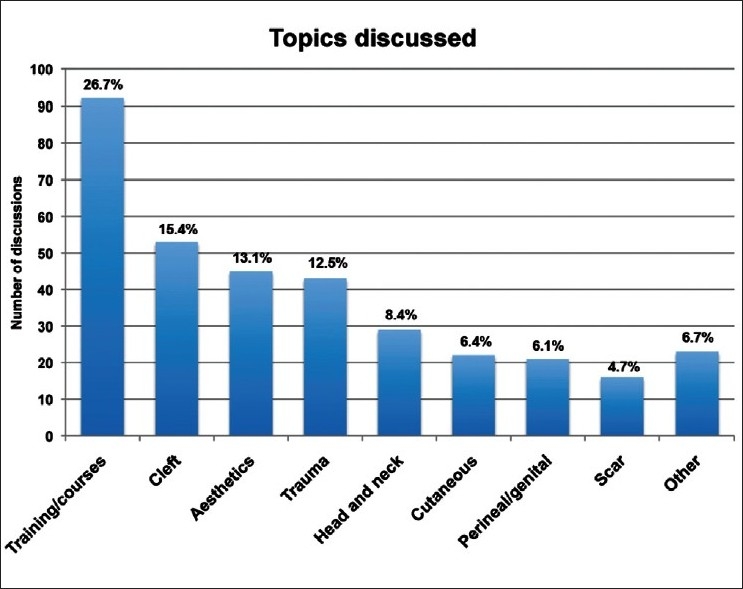
Topics discussed within the forum.

Discussions covered all areas of plastic surgery including training and professional development (92 threads), cleft (53 threads), aesthetics (45 threads), trauma (43 threads), head and neck (29 threads), cutaneous (22 threads), perineal/genital recontruction (21 threads), and scar management (16 threads) [Figure 1]. Fifty percent of discussions contained images. On average, there were five images per thread (range = 0–34). These included clinical photographs, radiographs, and drawings

## DISCUSSION

In 1998, John Chambers, then president of Cisco Systems, said “The Internet will change how people live, work, play and learn. The Industrial Revolution brought people together with machines in factories, and the Internet Revolution will bring together people with knowledge and information in virtual communities”.[1] Over 10 years later, his words still ring true.

The day after graduating from medical school in 1998, Dr. Sunil Soni joined his father’s plastic surgery practice in the then small town of Hisar in Haryana, India. The Internet arrived in Haryana a year later and Dr Soni soon stumbled upon a mailing list of orthopaedic surgeons. He saw the potential this type of group has to help like-minded individuals share ideas, knowledge and wisdom. Yahoogroups were launched in 2000 and in February 2001, plastic_surgery@yahoogroups was founded by Dr Soni.

Within 4 months, lively discussions were taking place. In the first 12 months, there were 497 messages. The activity rapidly grew and in 2009, there were 3211 messages, representing a greater than sixfold increase.

Members come from across the globe although a significant proportion comprises plastic surgeons practicing in India. They include every subspecialty and all levels of expertise. Needless to say, they bring with them a huge body of experience.

The wide range of topics discussed in this group means that all areas within plastic surgery – reconstructive, burns, hand, cranio-maxillofacial, aesthetic and onco-plastic are all well represented. Conditions considered rare in one part of the world can be addressed by those with experience of the condition from elsewhere on the globe, e.g. Dupuytren’s contracture, lymphatic filariasis.

The most common type of discussion is one where the originator posts a difficult problem he/she is faced with, asking for advice or ideas. There are always ample helpful suggestions. Occasionally, some will warn against a particular approach, having tried it unsuccessfully in the past. This pool of advice and ideas means that even practitioners working in remote locations need not feel alone or worry about having to fend for themselves. A vast majority of Indians still live in villages and travelling to centres of excellence is neither affordable nor possible. This discussion group is like a continueing medical education for the plastic surgeon in these remote regions so that they can deliver the state of art surgery there and thus render a geographical neutrality in terms of standards of surgery.

Members often post case reports highlighting novel techniques, simple tips/tricks, complicated cases or unexpected complications. These are not simply plastic surgeons “showing off” what they have achieved. Many highlight problems encountered and are accompanied by advice to avoid a particular approach so that similar errors are not repeated.

Half of all discussions contain images. Their liberal use helps illustrate the point. Additionally, when posting a reply, contributors often draw or annotate on a photograph posted by the originator to aid description of his/her suggestion or opinion.

No evidence for written consent for the use of these images exists. Perhaps this is one area that warrants attention. In countries like the UK and USA, written consent is standard practice prior to taking clinical photographs or performing invasive procedures. The process of obtaining informed consent allows risks, benefits and alternatives to be discussed with the patient. The opportunity can be taken to provide written information to the patient, an action which has been shown to improve patient satisfaction.[2] Although a signed consent form is not strictly a legal document, it serves as evidence that these areas have been discussed. With increasing exposure to medical practice abroad, better education and rising expectations, it is only a matter of time before informed written consent for such discussions becomes mandatory in India. It would be worth introducing standardised consent forms for clinical photographs and clinical discussions between specialists just as there are for clinical procedures. Most of the patients would feel privileged that their surgeon is making an extra effort to discuss their peculiar problem with his / her peers and then making a final decision regarding the nature of surgery. However if the results of surgery are not up to the satisfaction of the patient, then knowing that the discussion group had members with a different opinion on the subject may also lead to unwanted litigations. So while a consent from the patient may be useful, the discussions about them should be sacrosanct and impregnable by anyone outside the group.

There is a section where members can upload useful resources. Currently it is poorly populated, with only 17 files uploaded since the group began. This area is ideal for uploading a range of useful resources, e.g. patient information leaflets, lists of useful webpages, educational articles. Needless to say, it is imperative that copyright is not breached. Greater input here will benefit the group significantly.

A potential addition could be the development of an organised archiving system to collate previous messages and threads. A similar database could be set up for photographs, radiographs and powerpoint or pdf files. This will ease searching for information. Realistically, organising and categorising the 18,000 messages that have been exchanged in the last 9 years will be an almost insurmountable task. However, if done prospectively, this can be a useful addition.

In the 3 years that I have been a member of this group, I have certainly learnt and gained a lot. The ethos of serving the specialty, its practitioners and the patients remains the focus at all times. The fact this group continues to generate such interesting discussion and stimulating debate is a tribute to its members and an asset to the specialty. It is our responsibility as members to keep up the good work, and to further improve on this resource in whatever way possible.
